# Exploring the Susceptibility to Multiple Primary Tumors in Patients with Differentiated Thyroid Cancer

**DOI:** 10.3390/diagnostics14121210

**Published:** 2024-06-07

**Authors:** Laura Valerio, Silvia Cantara, Elisa Mattii, Cristina Dalmiglio, Alfonso Sagnella, Antonia Salvemini, Alessandra Cartocci, Fabio Maino, Maria Grazia Castagna

**Affiliations:** 1 Department of Medical, Surgical and Neurological Sciences, University of Siena, 53100 Siena, Italy; laura.valerio@ao-siena.toscana.it (L.V.); cantara@unisi.it (S.C.); e.matti@student.unisi.it (E.M.); cristina.dalmiglio2@unisi.it (C.D.); alfonso.sagnella@student.unisi.it (A.S.); antonia.salvemini@student.unisi.it (A.S.); fabio.maino@ao-siena.toscana.it (F.M.); 2 Department of Medical Biotechnologies, University of Siena, 53100 Siena, Italy; alessandra.cartocci@dbm.unisi.it

**Keywords:** thyroid cancer, relative telomere length, multiple primary tumors, prognostic risk factors

## Abstract

Purpose: It was demonstrated that differentiated thyroid cancer (DTC) patients may develop multiple primary tumors (MPT) during follow-up. Many studies showed an association between reduced telomere length and cancer phenotype; in particular, the short telomeres were associated with the development of a primary tumor. However, the role of altered telomere length in MPT development has not yet been demonstrated. The aim of this study was to evaluate the possible correlation between a short telomere length in blood leukocytes and the risk of developing MPT in DTC patients. Patients and Methods: We retrospectively evaluated 167 DTC patients followed up for a median of 13.6 years. Our control group was represented by 105 healthy subjects without any thyroid disease or present or past history of tumors. Our study groups, age-matched, were evaluated for the relative telomere length measured in leukocytes of peripheral venous blood. Results: The relative telomere length (RTL) was significantly different in healthy subjects compared to the total group of differentiated thyroid cancer patients [*p* < 0.0001]. Shorter telomeres length was observed in DTC patients with (*n* = 32) and without (*n* = 135) MPT compared to healthy subjects (*p* < 0.0001 and *p* = 0.0002, respectively). At multivariate analysis, the parameters independently associated with the presence of MPT were RTL [OR: 0.466 (0.226–0.817), *p* = 0.018] and the familial DTC [OR: 2.949 (1.142–8.466), *p* = 0.032]. Conclusions: The results of this study suggest a role of the relative telomere length in predicting MPT development in DTC patients. Our results contribute to increasing the knowledge of the genetic mechanisms underlying MPT development in DTC patients, considering relative telomere length as a possible prognostic marker.

## 1. Introduction

The occurrence of multiple primary tumors (MPT) in the same patient poses a significant clinical challenge warranting investigation in the field of oncology. These tumors, distinct from the initially diagnosed cancer, can independently arise in the same individual. Understanding the factors contributing to the development of MPT is crucial for patient-tailored management and care. Some studies demonstrated that oncological patients have an increased risk of developing MPT, and multiple factors can contribute to this risk [1,2,3,4,5,6,7].

The risk of MPT might be expected to be raised due to the persisting effects of genetic and lifestyle factors, environmental influences, and long-term side effects of previous chemo- and radiotherapy treatment [2,3].

Regarding the genetic factors, its known that genomic instability may contribute to increase MPT risk. Many studies demonstrated an association between altered telomere length, involved in the cell replicative cycle, and cancer development.

Telomeres are specific DNA structures at the end of chromosomes made up of repetitive nucleotide sequences. This, along with protein shelter, aids in maintaining chromosome stability and shielding them from genomic erosion.

It was demonstrated that both short and long telomere lengths have been associated with a high risk of cancer incidence. In particular, the majority of retrospective studies report an increased risk of cancer in individuals carrying shorter telomeres in blood leukocytes [8,9,10,11,12,13,14,15,16,17,18] but, in our knowledge, the role of shortened telomere length in MPT development has not yet been demonstrated.

Differentiated thyroid cancer (DTC) is generally characterized by a good prognosis, but in DTC patients, the risk of MPT must be considered [19,20,21,22]. In a recent study, we confirmed a higher risk of developing a second malignant tumor in non-medullary thyroid cancer patients (NMTC), and we demonstrated, for the first time, that patients with the familial form of NMTC showed a higher risk of developing a second malignant tumor compared to sporadic form of NMTC [23]. Moreover, our group demonstrated that patients with the familial form of NMTC showed shorter telomeres in the blood leukocytes compared to sporadic forms, nodular goiter, and healthy subjects [24]. On this basis, we hypothesized that the increased incidence of a second malignant tumor in the familial form of NMTC could be caused by a genetic factor such as the presence of shorter telomeres and genomic instability.

The aim of this study was to evaluate the possible correlation between a short telomere length in blood leukocytes and the risk of developing MPT in DTC patients in order to increase the knowledge of the genetic mechanisms underlying MPT development in DTC patients.

## 2. Materials and Methods

### 2.1. Study Group

We retrospectively evaluated 167 DTC patients surgically treated between May 1994 and October 2022 and followed up at our Institution for a median of 13.6 years (mean 13.3 ± 5.6 years, range 1.1–27.9 years). In our study group, 126 patients were females (75.5%), and 41 patients were males (24.5%), with a median age at DTC diagnosis of 44 years (mean 47.1 ± 17.4 years, range 13–85 years). Our control group was represented by 105 healthy subjects (33 females and 72 males; mean age 47 ± 12.5 years, range 32–71 years) without any thyroid disease or present or past history of tumors.

The study group and control group were matched for age.

Our study groups (DTC patients and control group) were characterized by determining the relative telomeres length in leukocytes of peripheral venous blood at the time of sample collection.

### 2.2. Measurement of Relative Telomere Length (RTL)

Ten milliliters of peripheral venous blood were obtained at the time of the study after the acquisition of signed informed consent. Genomic DNA was extracted with a salting out procedure, and DNA concentration was assessed by Nanodrop (Thermo Scientific, Milan, Italy). Measurement of RTL was assessed with quantitative PCR on 30 ng/µL using an MJ mini personal thermal cycler (BioRad, Milan, Italy) as already described [25]. Briefly, telomere length quantification involved determining the relative ratio of telomere repeat copy number to a single-copy gene copy number (the 36B4 gene) in experimental samples using standard curves. This ratio is proportional to the average telomere length. Primers and conditions are detailed in the Cawthon et al. study protocol [25].

### 2.3. Definitions of Multiple Primary Tumors (MPT)

MPT were defined as any primary malignancy with histological confirmation occurring at an anatomical site other than the thyroid. According to the timing of MPT occurrence with respect to DTC diagnosis, we divided the MPT group into pre-DTC (non-metachronous) and post-DTC (metachronous), based on the tumor diagnosis made at least 12 months before or after DTC, respectively. The MPT diagnosed within this period was considered synchronous.

### 2.4. Statistical Analysis

Data were presented as mean ± SD or median when needed or as absolute frequencies and percentages. The T-test for independent data or the Mann–Whitney U test were performed for variables, normally or non-normally distributed (evaluated by Kolmogorov–Smirnov), respectively. To evaluate significant differences in data frequency, the Chi-squared test, the Fisher exact test, or its approximation were performed according to the dimension of the contingency table and to the expected frequencies. Logistic regression was used to estimate the effects of several clinical and pathological features (gender, age at thyroid cancer diagnosis, familial/non-familial DTC, telomeres length) on the risk of MPT in DTC patients.

Statistical analysis was performed using the SPSS Statistics version 22.0. A *p*-value < 0.05 was considered statistically significant.

## 3. Results

### 3.1. Multiple Primary Tumors in the Study Group

In the study group, 32/167 DTC patients (19.2%) were affected by at least another primary tumor developed before or after DTC diagnosis. The MPT were non-metachronous in the majority of cases (67.8%), and the most common tumor was represented by breast cancer (40.6%), followed by lung cancer (12.5%), hematologic, kidney and skin malignancies (9.3%), respectively, colorectal cancer (6.2%), and other cancers in 12.8% of patients. In Table 1, we reported the clinical and pathological features of the two subgroups of patients, age-matched, defined according to the presence/absence of MPT. A longer follow-up was observed in the group without MPT compared to the group with MPT (median 14.4 years versus 10.0 years, respectively, *p* = 0.002). No differences between the two groups were observed regarding sex (*p* = 0.17), familial DTC (*p* = 0.99), ATA risk class at DTC diagnosis (*p* = 0.58), and clinical outcome at last follow-up (*p* = 0.70)

### 3.2. Association between RTL and Multiple Primary Tumors

**Figure 1:**
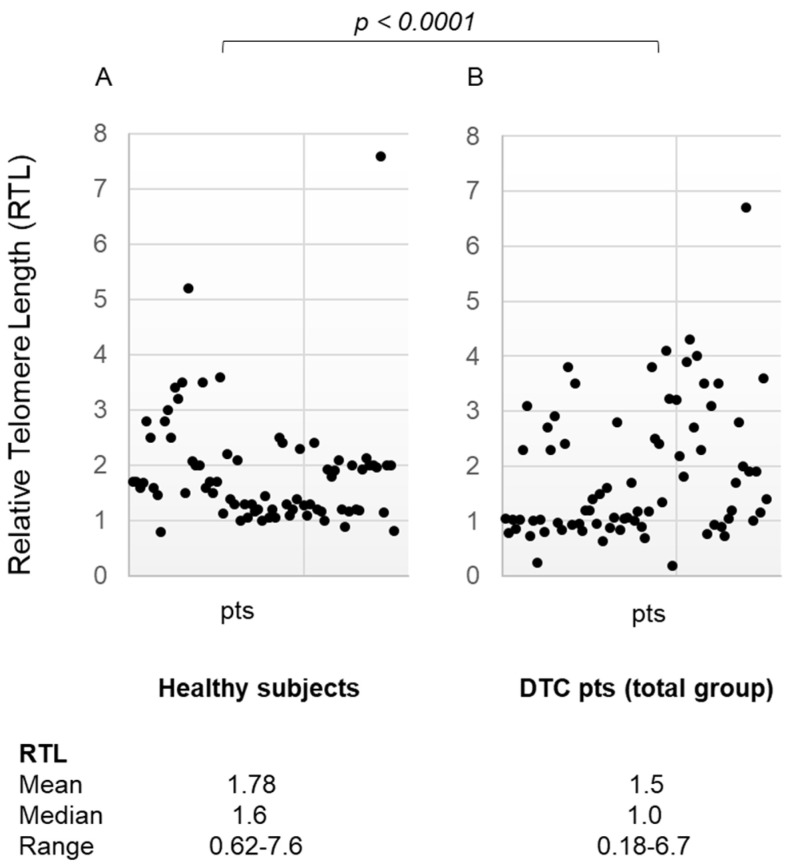
(panel (**A**)) Relative Telomere Length in the healthy subject group (media ± SD, median, range); (panel (**B**)) Relative Telomere Length in differentiated thyroid cancer patients (total group) (media ± SD, median, range). RTL: Relative Telomere Length; DTC: Differentiated Thyroid Cancer; MPT: Multiple Primary Tumors; pts: patients.

The age-adjusted relative telomere length (RTL) in blood leukocytes was significantly different in healthy subjects compared to the total group of DTC patients [mean ± SD 1.78 ± 0.93, median 1.6, range 0.62–7.6 in healthy subjects and mean ± SD 1.5 ± 1.0, median 1.0, range 0.18–6.7 in DTC patients, *p* < 0.0001] (Figure 1, panel A,B).

**Figure 2:**
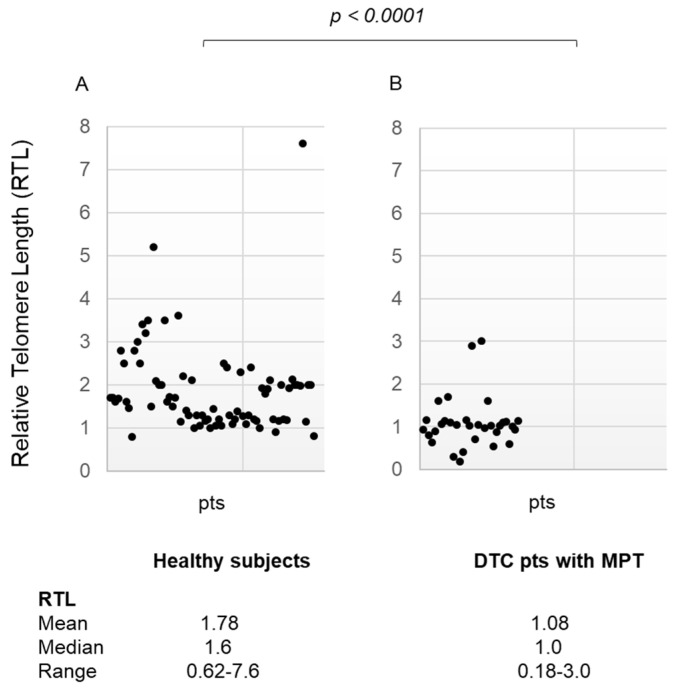
(panel (**A**)) Relative Telomere Length in the healthy subject group (media ± SD, median, range); (panel (**B**)) Relative Telomere Length in differentiated thyroid cancer patients with multiple primary tumors (media ± SD, median, range). RTL: Relative Telomere Length; DTC: Differentiated Thyroid Cancer; MPT: Multiple Primary Tumors; pts: patients.

**Figure 3:**
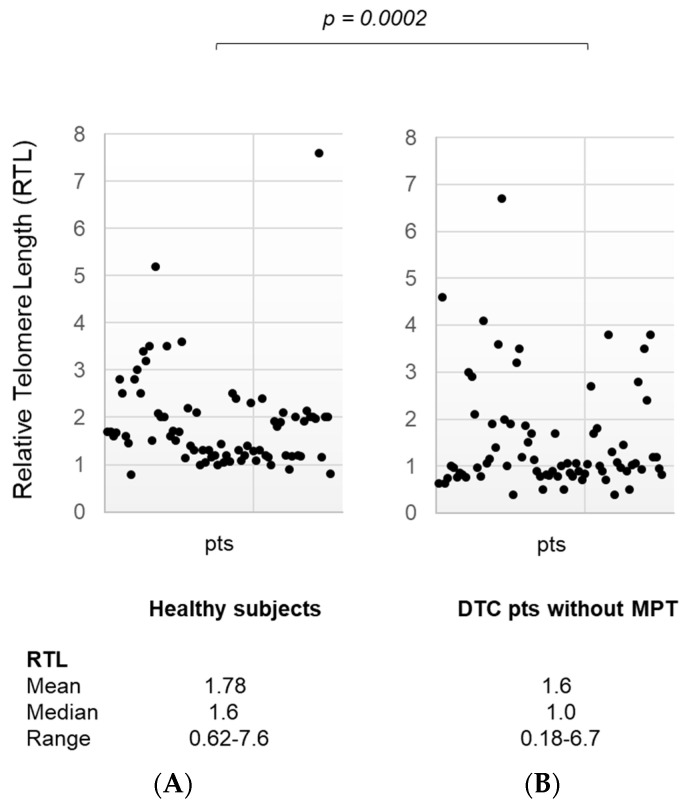
(panel (**A**)) Relative Telomere Length in the healthy subject group (media ± SD, median, range); (panel (**B**)) Relative Telomere Length in differentiated thyroid cancer patients without multiple primary tumors (media ± SD, median, range). RTL: Relative Telomere Length; DTC: Differentiated Thyroid Cancer; MPT: Multiple Primary Tumors; pts: patients.

When the same analysis was performed, comparing healthy subjects to DTC patients with (*n* = 32) or without (*n* = 135) MPT independently, RTL was significantly shorter in the subgroup of patients with MPT [mean ± SD 1.78 ± 0.93, median 1.6, range 0.62–7.6 in healthy subjects, and mean ± SD 1.08 ± 0.59, median 1.0, range 0.18–3.0 in DTC patients with MPT, *p* < 0.0001] (Figure 2, panel A,B) and also in DTC patients without MPT [mean ± SD 1.78 ± 0.93, median 1.6, range 0.62–7.6 in healthy subjects, and mean ± SD 1.60 ± 1.1, median 1.0, range 0.18–6.7 in DTC patients without MPT, *p* = 0.0002] (Figure 3, panel A,B).

**Figure 4:**
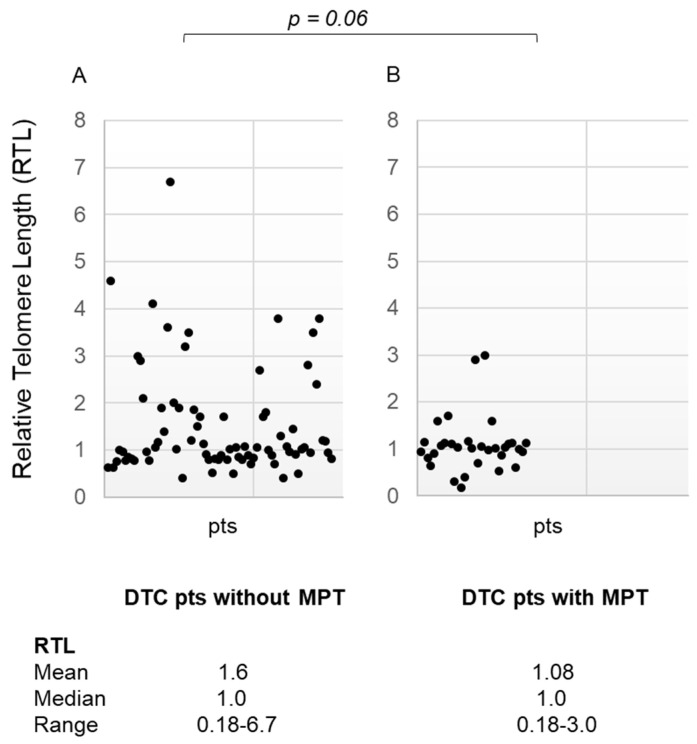
(panel (**A**)) Relative Telomere Length in differentiated thyroid cancer patients without multiple primary tumors (media ± SD, median, range); (panel (**B**)) Relative Telomere Length in differentiated thyroid cancer patients with multiple primary tumors (media ± SD, median, range). RTL: Relative Telomere Length; DTC: Differentiated Thyroid Cancer; MPT: Multiple Primary Tumors; pts: patients.

When relative telomere length was compared between the two subgroups of DTC patients (with and without MPT), we observed that the relative telomere length was shorter in DTC patients with MPT, with a difference very closely to statistical significance (*p* = 0.06) (Figure 4, panel A,B).

### 3.3. Predictive Factors of MPT in DTC Patients

In order to identify the possible predictors of MPT in DTC patients, we performed a multivariate analysis including several parameters that may be involved in the development of MPT (age at DTC diagnosis, sex, familial or sporadic form of DTC, and the relative telomere length). The parameters independently associated with the presence of MPT were the relative telomeres length [OR: 0.466 (0.226–0.817), *p* = 0.018] and the familial DTC [OR: 2.949 (1.142–8.466), *p* = 0.032].

## 4. Discussion

In the field of oncology, it is important to investigate the clinical challenge of MPT in the same patient. Unlike the initially diagnosed cancer, these tumors can develop independently in the same individual. The development of MPT requires an understanding of the factors that contribute to it in order to provide patient-tailored management and care. Some studies have shown that oncological patients are at a higher risk of developing MPT, and these risks can be influenced by multiple factors (i.e., genetic and lifestyle factors, environmental influences, and long-term side effects of previous chemo- and radiotherapy treatment) [1,2,3,4,5,6,7].

Regarding genetic factors, an important role of altered telomere length in the development of primary malignancies has been demonstrated [8,9,10,11,12,13,14,15,16]. Telomeres are specialized structures at the ends of chromosomes, consisting of hundreds of repeated hexanucleotides (TTAGGG)n. Genetic integrity is partly maintained by the architecture of telomeres, and it is gradually lost as telomeres progressively shorten with each cell replication due to incomplete lagging DNA strand synthesis and oxidative damage [26,27].

In this study, we hypothesized that the presence of MPT in DTC patients may be associated with shortened telomeres in blood leukocytes. Our study showed that MPT were present in 19.2% of DTC patients, either developed before or after the diagnosis of thyroid cancer. In our study group, the MPT were mainly non-metachronous (67.8%), and breast cancer was the most frequent malignancy (40.6%), followed by lung cancer (12.5%), hematologic, kidney, and skin malignancies (9.3%), respectively, colon malignancy (6.2%), and other MPT in 12.8% of patients.

The evaluation of clinical and pathological features of DTC patients with or without MPT showed no differences between the two groups. We evaluated the relative telomere length in blood leukocytes of our patients, and when compared DTC patients, with and without MPT, to healthy subjects, we observed that both groups of DTC patients had a shorter telomere length than healthy subjects (*p* < 0.0001 and *p* = 0.0002, respectively).

In humans, it has been reported that females have longer telomeres than males and that this association becomes stronger with increasing age. This difference is believed to be influenced by a variety of biological factors, including hormonal differences, genetic factors, and lifestyle behaviors. Estrogen, for instance, is thought to have a protective effect on telomeres, potentially contributing to the longer telomere length observed in females [28]. Understanding these differences is important for interpreting telomere length data in clinical and research settings. To avoid this possible confounding factor in our results, we performed a statistical analysis of males and females separately. Significantly shorter RTL was documented in males with DTC when compared with healthy males (*p* = 0.0004); similarly, shorter RTL was documented in females with DTC when compared with healthy females (*p* = 0.007).

Moreover, when we compared the two subgroups of DTC patients (with and without MPT), the subgroup with MPT showed shorter telomeres than DTC patients without MPT, approaching statistical significance (*p* = 0.06). We speculated that these results might be attributed to the small cohort of DTC patients. It is possible that by increasing the number of patients with DTC, a significant difference between DTC patients with and without MPT could be documented.

In the literature, some studies demonstrated that oncological patients have an increased risk of developing MPT, and multiple factors can contribute to this risk [1,2,3,4,5,6,7] (persisting effects of genetic and lifestyle factors, environmental I nfluences, and long-term side effects of previous chemo- and radiotherapy treatment) [2,3]. Only a few studies demonstrated the correlation between telomere length and the risk of a second tumor in childhood cancer survivors, suggesting that shorter telomeres may contribute to developing a second primary tumor in these patients [29,30,31]. However, to our knowledge, the role of altered telomere length in MPT development has not yet been demonstrated.

In our series, to identify the possible predictors of MPT in DTC patients, we performed a multivariate analysis including several parameters that may be involved in the development of MPT (age, sex, familial or sporadic form of DTC, and the relative telomere length). The analysis showed that the parameters independently associated with the presence of MPT were the relative telomeres length [OR: 0.466 (0.226–0.817), *p* = 0.018] and the familial DTC [OR: 2.949 (1.142–8.466), *p* = 0.032]. These results demonstrate that an increase of one unit in telomere length reduces the risk of having MPT in DTC patients by 56% [OR: 0.466 (0.226–0.817)]. Moreover, the correlation between familial DTC and the presence of MPT confirms our previous results that showed, for the first time, that patients with familial DTC have a higher risk of developing a second malignant tumor compared to patients with sporadic forms of DTC. We concluded that different cancers share common genetic factors that, probably in association with environmental factors, contribute to an increase in the relative risk for the same or other cancers beyond the nuclear family [23].

The present study has limitations and strengths. The limits of our study include the lack of baseline RTL evaluated before DTC diagnosis and treatment and the impossibility of evaluating the RTL during follow-up. Furthermore, due to the retrospective nature of this study, data concerning alcohol consumption and tobacco use, both known to shorten telomeres and implicated in the development of various cancers, were unavailable.

The strength includes the longest follow-up of the patients in the same Institute, which allows us to have detailed information regarding DTC diagnosis, treatment, outcome, and diagnosis of MPT before or after DTC.

## 5. Conclusions

In conclusion, this is the first study that suggests the role of the relative telomere length in predicting the development of MPT in DTC patients. Moreover, our results suggest that genomic instability and familial DTC play an important role in the development of MPT in DTC patients. These results may contribute to increasing the knowledge of the genetic mechanisms underlying MPT development in DTC patients, considering the relative telomere length as a prognostic marker. Further studies are needed to validate these results in order to tailor the management and care of these patients.

## Figures and Tables

**Table 1:** Clinical and pathological features of DTC patients according to the presence/absence of multiple primary tumors.

	Multiple Primary Tumors		
	Yes (*n* = 32)	No (*n* = 135)	*p*-Value
Sex (*n*, %)			
Male	11 (34.4%)	30 (22.2%)	0.15
Female	21 (65.6%)	105 (77.8%)	
Familial differentiated thyroid cancer			
Yes	15 (46.9%)	63 (46.7%)	0.99
No	17 (53.1%)	72 (53.3%)	
Papillary thyroid cancer	31 (96.9%)	133 (98.5)	0.06
Follicular thyroid cancer	1 (3.1%)	2 (1.5%)	
ATA risk class (*n*, %)			
Low	17 (53.1%)	80 (59.3%)	0.58
Intermediate	12 (37.5%)	47 (34.8%)	
High	3 (9.4%)	8 (5.9%)	
Total thyroidectomy and radioiodine treatment:			
Low risk	9 (39.1%) *	64 (53.8%) ^#^	0.06
Intermediate risk	11 (47.8%)	47 (39.5%)	
High risk	3 (13.1%)	8 (6.7%)	
Clinical outcome (*n*, %)			
Excellent response	24 (75.1%)	110 (81.5%)	0.7
Indeterminate Response	4 (12.5%)	14 (10.4%)	
Biochemical incomplete response	2 (6.2%)	6 (4.4%)	
Structural incomplete response	2 (6.2%)	5 (3.7%)	
Follow-up (years)			
Median	10	14.4	0.002
Mean ± SD	10.6 ± −5.5	14 ± 5.5	
Range	2.9–23.3	1.2–27.9	

* In DTC pts with MPT, nine low-risk pts were not treated with radioiodine therapy. ^#^ In DTC pts without MPT, 16 low-risk pts were not treated with radioiodine therapy.

## Data Availability

The data presented in this study are available on request from the corresponding author. The data are not publicly available due to patient privacy.
